# Carbamazepine-induced Red Blood Cell Aplasia: A Case Report

**DOI:** 10.5505/tjh.2012.52296

**Published:** 2012-06-15

**Authors:** Halit Özkaya, Gökhan Aydemir, Abdullah Barış Akcan, Mustafa Kul, Ferhan Karademir, Seçil Aydınöz, Selami Süleymanoğlu

**Affiliations:** 1 Gata Haydarpaşa Teaching Hospital, Department of Pediatrics, Uskudar, Istanbul, Turkey

## TO THE EDITOR

A 2-year-old boy developed pure red cell aplasia (PRCA) during carbamazepine (CBZ) therapy. There was no history of prolonged or profuse bleeding after cuts or injury, and no bleeding from any sites of the body, petechiae, ecchymosis, or bruising. His family history of hematological disorders was negative. The child had been receiving CBZ therapy at a daily dose of 10 mg kg^–1^ for the last 6 months due to generalized tonic clonic seizures. One month before starting CBZ the boy’s complete blood count was analyzed due to anorexia and the findings were normal. 

The boy was 2 years old when he was admitted to our hospital due to lower respiratory tract infection and severe anemia was then noted . On physical examination he was pale and showed no signs of lymphadenopathy, hepatosplenomegaly, jaundice, or petechiae. Rhonchi and crackles were noted on auscultation of the lungs. The patient was diagnosed as pneumonia and 2nd-generation cephalosporin treatment was initiated. The patient’s laboratory findings on admission were as follows: red blood cell count: 150 x 10^4^ L^–1^; white blood cell count: 11,000 L^[u]–1[/u]^; hemoglobin concentration: 4.5 g dL^–1^; hematocrit: 13%; platelet count: 156 x 10^4^ L^–1^; reticulocytes: 0.2%; MCV: 75 fL; MCH: 22.1 pg; MCHC: 28.1 g/dL. The serum iron level was 60 g dL^–1^ and total iron binding capacity was 250 g dL^–1^. Hepatitis B antigen was negative, whereas antibodies were positive. Direct and indirect Coombs test results were negative. Serum ferritin, vitamin B12, folic acid, and bilirubin were all within the normal range. The patient’s serum CBZ concentration was 6 μg mL–1. Analysis of bone marrow aspirate showed cellular marrow with normal granulopoiesis and megakaryocytopoiesis, but the almost complete absence of erythroid precursor cells. The possibility of CBZ-induced PRCA was considered and CBZ was withdrawn on d 2 of hospitalization. The patient required transfusion of packed red cells. Two weeks after CBZ was withdrawn the patient had a rapid reticulocyte response. 

Analysis of bone marrow aspirate during the recovery phase showed normal erythropoiesis and a myeloid:erythroid ratio of 1:1. PRCA resolved following discontinuation of CBZ. The patient’s serial hemogram results are shown in the Table 1. 

Although the hematological toxicity of CBZ is well documented, isolated cessation of red cell production is uncommon [1]. Most of the known adverse effects of CBZ are usually transient and dose-related; however, hematological side effects are the most serious and are often not dose-related [2,3]. PRCA is an uncommon complication of CBZ therapy [4,5,6,7,8]. PRCA exists in several forms, and the most common is an acute self-limited condition [10] that is secondary to virus- and drug-induced impairment of erythroid progenitor cells [9,11]. Acquired PRCA in childhood is classified as primary or secondary. Most cases of childhood primary acquired PRCA include transient erythroblastopenia of childhood (TEC). The causes of secondary acquired PRCA include infections, autoimmune reactions, tumors, drugs, and toxins [3]. Viral causes of PRCA are parvovirus B19, hepatitis virus (hepatitis A and C), Epstein-Barr virus, cytomegalovirus,varicella-zoster virus, human immunodeficiency virus. In the presented patient PRCA was not induced by a virus. Some antiepileptic drugs have been implicated in the development of PRCA, including diphenylhydantoin, sodium valproate, and CBZ. The exact mechanism of erythropoietic suppression exerted by these drugs remains unclear. 

This presented case shows that hematological toxicity can occur in response to CBZ treatment, indicating that patient’s treated with CBZ must be monitored closely and regularly; in such patients any decrease in the hemoglobin level requires careful monitoring for the sudden development of serious anemia. 

**Conflict of Interest Statement **

None of the authors have any conflicts of interest, including specific financial interests, relationships, and/ or affiliations, relevant to the subject matter or materials included in this manuscript. Informed consent for publication was obtained from the patient’s parents.

## Figures and Tables

**Table 1:**
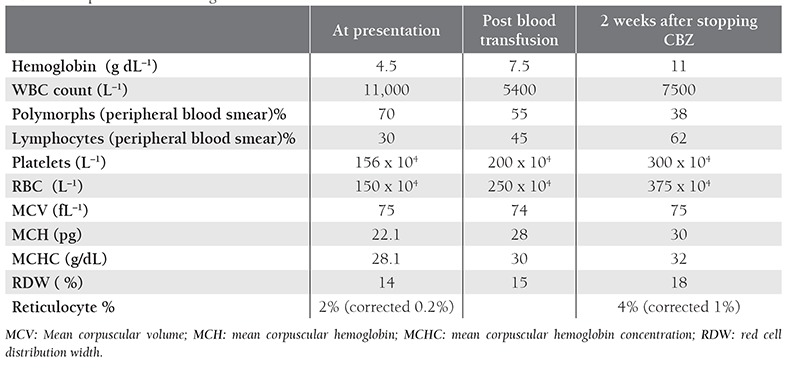
The patient’s serial hemogram results.
